# ﻿*Isotremaputalengense*, a new species of Aristolochiaceae from northern Vietnam and two new combinations in *Isotrema*

**DOI:** 10.3897/phytokeys.197.73596

**Published:** 2022-05-27

**Authors:** Quoc Binh Nguyen, Hieu Cuong Nguyen, Duc Binh Tran, Phuong Hanh Nguyen, Hong Truong Luu

**Affiliations:** 1 Vietnam National Museum of Nature, Vietnam Academy of Science and Technology, 18 Hoang Quoc Viet Street, Cau Giay District, Hanoi, Vietnam Vietnam National Museum of Nature, Vietnam Academy of Science and Technology Hanoi Vietnam; 2 Southern Institute of Ecology, Institute of Applied Materials Science and Graduate University of Science and Technology, Vietnam Academy of Science and Technology, 1D, TL29 Street, District 12, Ho Chi Minh City, Vietnam Science and Graduate University of Science and Technology Ho Chi Minh Vietnam; 3 Institute of Ecology and Biological Resource, Vietnam Academy of Science and Technology, 18 Hoang Quoc Viet Street, Cau Giay District, Hanoi, Vietnam Institute of Ecology and Biological Resource, Vietnam Academy of Science and Technology Hanoi Vietnam

**Keywords:** *Aristolochia*, *Isotremavuquangense*, *Isotremayachangense*, Pu Ta Leng Mountain, *Siphisia*

## Abstract

*Isotremaputalengense* Luu, Q.B.Nguyen & H.C.Nguyen is described as a new species from northern Vietnam. It looks most morphologically like *I.wardianum* but is distinguishable by a combination of different leafy and floral characters. Morphological comparison between the new plant and closest species is provided. In addition, combinations of two recently described *Aristolochia* species are made, namely *Isotremavuquangense* (T.V.Do) Luu, Q.B.Nguyen & H.C.Nguyen and *Isotremayachangense* (B.G.Huang, Yan Liu & Y.S.Huang) Luu, Q.B.Nguyen & H.C.Nguyen.

## ﻿Introduction

*Isotrema* Raf. (Aristolochiaceae) was recently resurrected as a genus independent from *Aristolochia* L. (Zhu et al. 2019a). Species of *Isotrema* are, in fact, those of Aristolochia subgenus Siphisia (Duch.) O.C.Schmidt (Schmidt 1935) and differ from others of *Aristolochia* by having strongly curved perianth, 3-lobed gynostemium, anthers paired on the outer surface of each gynostemium segment, and basipetally dehiscing capsule. This generic concept is followed in many later publications (Li et al. 2019; Zhou et al. 2019; Zhu et al. 2019b, c, d; Cai et al. 2020a; Wang et al. 2020a, b). Although several other authors still prefer assigning their newly described species under Aristolochia subgenus Siphisia (e.g., Cai et al. 2020b; Luo et al. 2020; Zhou et al. 2020; Do et al. 2021), of which *Isotrema* was accepted as one of the synonyms in the most recent nomenclatural review of *Aristolochia*-related taxa by Ohi-Toma and Murata (2016), the phylogenetic results by Zhu et al. (2019a) appear to be robust because of their extensive samples of Asian species and combination of molecular, chromosomic and morphological data. Therefore, *Isotrema* is followed in this paper.

To date, more than one hundred *Isotrema* species have been reported, including those named under *Aristolochia* (e.g., Liu and Deng 2009; Xu et al. 2011; Yao 2012; Huang et al. 2013; Wu et al. 2013; Do et al. 2014; Lu and Wang 2014; Nguyen et al. 2014; Ohi-Toma et al. 2014; Huang et al. 2015; Wu et al. 2015; Zhu et al. 2015; Do et al. 2016; Do et al. 2017; Do et al. 2018; Zhu et al. 2018; Zhou et al. 2019; Zhu et al. 2019a; Cai et al. 2020a, b; Do et al. 2021). Prior to this paper, 18 *Isotrema* species have been recorded for Vietnam (Lecomte 1909; Schmidt 1935; Pham-hoang 2000; Do et al. 2014; Do et al. 2015a, b; Do et al. 2016; Do et al. 2017; Do and Li 2018; Lai et al. 2019; Do et al. 2021).

During our botanical surveys in Pu Ta Leng Mountain, Lai Chau Province of northern Vietnam in 2020, we encountered a species that looks very much like *I.wardianum* (J.S.Ma) X.X.Zhu, S.Liao & J.S.Ma from China, India and Myanmar (Ma 1989; Zhu et al. 2019a; Wang et al. 2020b). After careful examination of the plant, we concluded it is a new species that is described here. Terminology follows Hou (1984) and Do et al. (2015a).

## ﻿Taxonomy treatments

### ﻿ Isotrema putalengense

Taxon classificationPlantaeGentianalesApocynaceae

Luu, Q.B.Nguyen & H.C.Nguyen sp. nov.

7F8F1B6F-0D70-5CF0-8E80-FC6A71247CC2

urn:lsid:ipni.org:names:77298657-1

Fig. 1

#### Type.

Vietnam. Lai Chau Province, Tam Duong District, Pu Ta Leng Mountain, 22°27'17"N, 103°33'07"E, 2329 m elevation, 14 June 2020, *Nguyen Quoc Binh*, *Tran Duc Binh*, *Doan Hoang Son*, *Nguyen Hieu Cuong SH992* (holotype, VNMN!; isotypes, SGN!, VNMN!).

**Figure 1.**
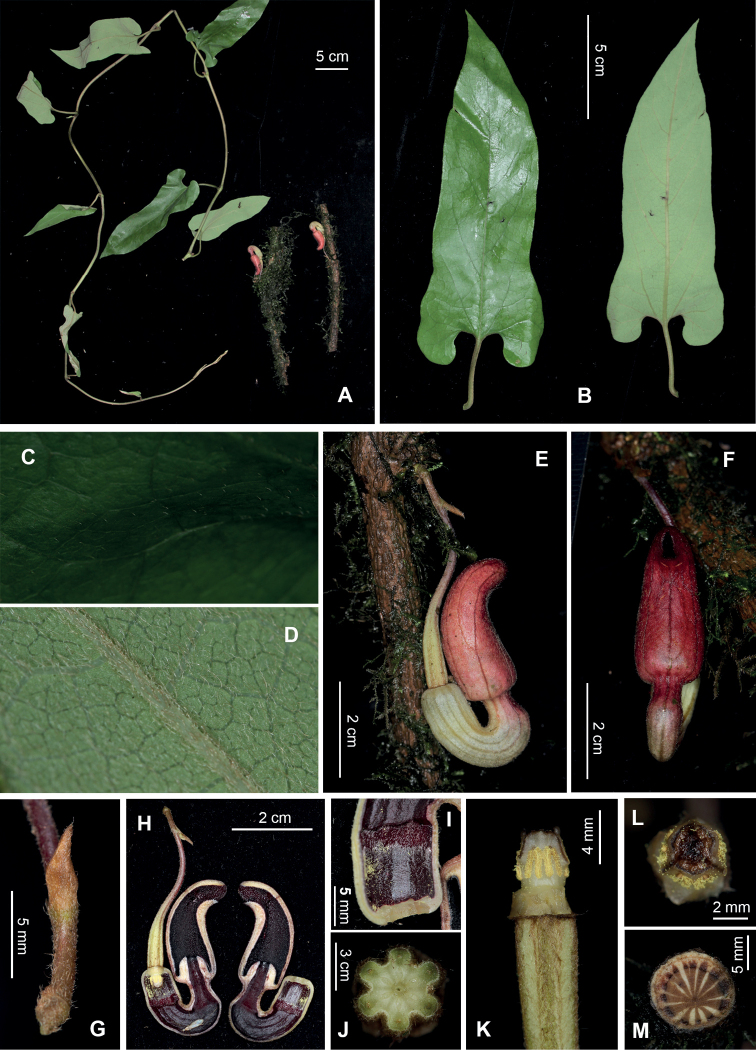
*Isotremaputalengense* Luu, Q.B.Nguyen & H.C.Nguyen **A** habit **B** leaf **C** leaf lamina, adaxial surface **D** leaf lamina, abaxial surface **E** flower, side view **F** flower, front view **G** bracteole **H** perianth, longitudinal dissection **I** utricle, inside **J** ovary, cross section **K** gynostemium, side view **L** stigma, view from above **M** stem, cross section. Photographs by Hieu Cuong Nguyen from *SH992* at the type locality.

#### Diagnosis.

The new species is most morphologically similar to *I.wardianum* in the shape of leaves and flowers but differs in having densely brown villous (vs. abaxially light brown villous) bracteoles, flowers on old woody stems (vs. in axils of leafy shoots), basally truncate perianth limb that is ovoid in front view and with purple apex (vs. basally obtuse, oblong in front view and with yellow apex), indistinct (vs. distinct) utricle from lower tube, U-shaped (vs. V-shaped) tube notch and internally black purple (vs. purple) tube.

#### Description.

Liana perennial, woody. Stems terete, pubescent. Petioles 3–4.5 cm long, densely pubescent; laminas lanceolate to slightly pandurate, 15–20 × 4–6 cm, adaxially sparsely pubescent, abaxially pubescent, margin entire, base auriculate, apex acute; veins palmate, 1 pair from base, lateral veins 3–4-paired; venation slightly adaxially sunken, abaxially prominent. Flowers on old woody stems, solitary; pedicel 2.5–3 cm, densely brown villous; bracteole inserted on basal half of pedicel, triangular, 5–5.5 mm long, 4–5.5 mm wide at base, densely brown villous, persistent. Ovary yellowish, 1.8–2.1 cm, 0.3–0.4 cm in diameter, densely brown villous, 6-ridged. Perianth horseshoe-shaped (in lateral view), 4–4.5 cm high, yellowish to purple, outside densely yellowish to brown hirsute with parallel veins, inside dark purple. Utricle indistinct from the tube, cylindrical, 7–9 mm long, 7–8 mm in diameter, outside light yellow, inside pilose and dark purple. Tube 3.5–4.0 cm, horseshoe-shaped, folded upwards at its middle forming a U-shaped notch, inside glabrous; lower tube 1.7–1.9 cm high and 0.6–0.7 cm in diameter, basally light yellow, apically purple; upper tube 0.6–0.7 cm long and 0.5–0.6 cm in diameter, parallel to the utricle, slightly constricted at the middle, purple; limb cylindric, ovoid in front view, curved forward, with truncate base, 2.5–2.7 cm long × 1.2–1.3 cm in diameter, inside dark red with dense dark-purple papillae, 3-lobed; lobes widely triangular, 0.5–1.3 mm high × 2–4 mm wide; throat ca. 3–4 mm high × 2 mm wide; annulus hemispherical, 0.5–0.6 cm high × 0.6–0.7 cm in diameter at base. Anthers 6, oblong, 2–2.2 mm long, adnate in 3 pairs to base of gynostemium. Gynostemium 3.5–4 mm long × 3.5–4 mm in diameter, stipitate; stipe ca. 0.5 mm; stigma connate, slightly 3-lobed; lobes (in older state) irregularly toothed. Fruits not seen.

#### Phenology.

Flowering found in June, fruiting unknown.

#### Etymology.

The specific epithet refers to the type locality, Pu Ta Leng Mountain which is part of the Hoang Lien Son Mountain Range and located about 30 km northwest of Vietnam’s highest Mt. Fan Si Pan.

#### Common and vernacular names.

Putaleng’s pipevine (Vietnamese name: Phòng kỷ Pu Ta Leng).

#### Distribution and habitat.

The new species is currently only known from Pu Ta Leng Mountain (with its highest peak at 3.049 m elevation), Tam Duong District, Lai Chau Province. It grows on humid fertile soils under a closed broadleaved evergreen forest unexplored botanically. There is no data available on the forest cover of the mountain. Our preliminary notes indicate that this forest is dominated by the Fagaceae, Lauraceae, Theaceae, Ericaceae and Magnoliaceae that are common families on the Hoang Lien Son Mountain Range, which is geographically considered part of the southern extension of the Himalayas and phytogeographically located in the Sikang-Yunnan Province (Averyanov et al. 2003).

#### Preliminary extinction risk assessment.

The plant was recorded in a small population with few scattered individuals in a presently unprotected large forest. It may be found in adjacent similar forests on the Hoang Lien Son Mountain Range. Given this fact, it is provisionally assigned as Data Deficient until more information is recorded (IUCN 2012; IUCN Standards and Petitions Committee 2022).

#### Discussion.

*Isotremaputalengense* is most morphologically similar to *I.wardianum* but they have a number of differences as expressed in the diagnosis. Besides, the new species is also close to *I.utriforme* (S.M.Hwang) X.X.Zhu, S.Liao & J.S.Ma (Hwang 1981; Zhu et al. 2019a) in the shape of leaves and flowers but the latter has glabrous and longer (4–8 cm) petiole, yellow-green flowers borne in axils of leafy shoots, ovate-lanceolate bracteoles inserted above middle of peduncle, short upper tube (3–4 mm), convex annulus, saccate limb with ovate-deltate and erect lobes. The shape of flowers in the new species looks like that in *I.pseudoutriforme* (X.X.Zhu & J.S.Ma) X.X.Zhu, Jun Wang & J.S.Ma and *I.ovatifolium* (S.M.Hwang) X.X.Zhu, S.Liao & J.S.Ma (Hwang 1981; Zhu et al. 2019a, e) but *I.pseudoutriforme* has ovate to narrowly ovate leaves and plain light yellow flowers, uncurved limb forming obtuse angle with upper tuber and ring-like annulus and *I.ovatifolium* has ovate leaves and abaxially densely off-white villous, purple-red flowers in axils of leafy shoots. The key morphological differences between the new species and those closest species are presented in Table 1.

**Table 1.** Morphological differences between *Isotremaputalengense* and close species (based on Hwang 1981; Ma 1989; Hwang et al. 2003; Zhu et al. 2019e; Wang et al. 2020b).

Characters	*I.putalengense*	*I.ovatifolium*	*I.pseudoutriforme*	*I.utriforme*	*I.wardianum*
Petiole	densely pubescent, 3–4.5 cm long	villous, 3–5 cm long	densely pubescent, 2–5 cm long	glabrous, 4–8 cm long	densely villous, 3–5 cm long
Lamina	lanceolate to slightly pandurate, 15–20 × 4–6 cm, with auriculate base, adaxially sparsely pubescent, abaxially pubescent	ovate, 5–13 × 4–8 cm, with cordate base, abaxially villous, adaxially glabrescent (densely villous when young)	ovate to narrowly ovate, 10–22 × 7–13 cm, with cordate base, adaxially sparsely pubescent, abaxially densely pubescent	ovate-lanceolate, 10–17 × 3–4 cm, with auriculate base, adaxially glabrous	lanceolate, 12–16 × 3–4 cm, with auriculate base, adaxially subglabrous to glabrous
Pedicel	2.5–3 cm long	3–6 cm long	1.8–5 cm long	4–6 cm long	1–2.5 cm long
Bracteoles	triangular, inserted on basal half of pedicel	ovate, inserted on basal 1/2 of pedicel	ovate, inserted on basal half and/or distal half of pedicel	ovate-lanceolate, inserted above distal half of pedicel	ovate, inserted on basal half of pedicel
Flower position	on old woody stems	axillary	axillary, sometimes on stems	axillary	axillary
Perianth limb	cylindric, ovoid in front view, straightly extended from upper tube, purple, 2.5–2.7 cm long × 1.2–1.3 cm in diameter, abaxially densely yellowish to brown hirsute	subcylindric, straightly extended from upper tube, purple-red, 1.5–2.5 cm long × 1–1.5 cm in diameter, abaxially densely off-white villous	cylindric, forming obtuse angle with upper tuber, light yellow, 2–3 cm long × 1–1.7 cm in diameter, abaxially sparsely villous	ovoid, straightly extended from upper tube, yellow-green, 1–2 cm long × ca. 1 cm in widest diameter, abaxially sparsely pilose to glabrous	cylindric, oblong in front view, forming obtuse angle with upper tube, purple with yellow apex, ca. 2.5 cm long × 0.9 cm in diameter, abaxially densely yellow villous
Limb lobes	wide triangle	subrounded or nearly truncate	triangle or wide triangle	ovate-deltate	wide triangle
Perianth throat	ca. 3–4 mm wide	ca. 2.5 mm wide	ca. 6 mm wide	ca. 1 mm wide	ca. 2–3 mm wide
Utricle	indistinct from lower tube, 7–8 mm in diameter, light yellow	indistinct from lower tube, 3–5 mm in diameter, purple-red	indistinct from lower tube, ca. 7–9 mm in diameter, light yellow	indistinct from lower tube, 3–4 mm in diameter, yellow-green	distinct from lower tube, 5 mm in diameter, light yellow
Tube notch	U-shaped	V-shaped	U-shaped	V-shaped	V-shaped
Upper tube	6–7 mm long × 5–6 mm in diameter, purple	ca. 3–5 mm long × 3–4 mm in diameter, purple-red	3–4 mm long × 6–8 mm in diameter, light yellow	ca. 3–5 mm long × 5–6 mm in diameter, yellow-green	ca. 10 mm long × 6 mm in diameter, purple
Annulus	hemispherical	flat	ring-like, raised	convex	hemispherical
Stigma lobes	truncate to slightly obtuse, irregularly toothed	obtuse, entire	round, entire	obtuse, entire	obtuse, entire

The leaves of the new species resemble those of *I.cucurbitoides* (C.F.Liang) X.X.Zhu, S.Liao & J.S.Ma (Liang 1975; Hwang et al. 2003; Zhu et al. 2019a) and *I.yangii* X.X.Zhu & J.S.Ma (Zhu et al. 2019e; Wang et al. 2020a) but these two species are readily different in a number of characters: *I.cucurbifoides* has leaves with 7–10 pairs of lateral veins, brownish flowers in axils of leafy shoots, ovate bracteoles, geniculately curved tube, 20 mm long utricle and deeply lobed perianth limb straight extended from upper tube and with 5–7 mm long lanceolate-acuminate lobes while *I.yangii* has leaves with 6–15-pairs of lateral veins, yellowish-white perianth with distinct purple stripes, 25–35 mm long utricles, internally smooth and pinkish or ochre perianth limb that is deeply 3-lobed and straight extended from upper tube and 16–24 mm long limb lobes.

Notably, the notch at the bent perianth tube of *I.putalengense* is obviously U-shaped while it is quite properly V-shaped in the above compared species except *I.pseudoutriforme* where the U-shaped notch is much narrower than that in the new species. Our field observations provisionally indicate that the notch shape is stable in, and could be typical for, *Isotrema* species. This character is more representative on longitudinal dissection of the perianth tube. However, its value as a supplemental taxonomic character for species identification has not been paid attention to in former *Isotrema* studies and needs further examination.

##### ﻿New combinations for some species of *Isotrema*

As a result of their study, Zhu et al. (2019a) has already transferred almost all species of Aristolochia subgenus Siphisia to *Isotrema*. Another four combinations were made for later described species (Wang et al. 2020a). Following this generic concept, here we propose new combinations for the other taxa of the subgenus that were described recently.

### ﻿ Isotrema vuquangense

Taxon classificationPlantaeGentianalesApocynaceae

(T.V.Do) Luu, Q.B.Nguyen & H.C.Nguyen comb. nov.

B0233A34-4FAC-55F3-BADF-38431BA6E3AD

urn:lsid:ipni.org:names:77298658-1

≡ Aristolochiavuquangensis T.V.Do. Phytotaxa 500 (1): 41. 2021.

#### Type.

Vietnam. Ha Tinh Province: Vu Quang District, Vu Quang National Park, 1103 m elevation, 18°15.133'N, 105°25.657'E, 30 August 2020, *Do Van Truong DVT379* (holotype VNMN; isotypes HN, VNMN).

### ﻿ Isotrema yachangense

Taxon classificationPlantaeGentianalesApocynaceae

(B.G.Huang, Yan Liu & Y.S.Huang) Luu, Q.B.Nguyen & H.C.Nguyen comb. nov.

4D032A6C-7455-5BC7-A1E2-9B1BB0FB480E

urn:lsid:ipni.org:names:77298659-1

≡ Aristolochiayachangensis B.G.Huang, Yan Liu & Y.S.Huang. PhytoKeys 153: 51. 2020.

#### Type.

China. Guangxi Zhuang Autonomous Region: Baise City, Leye County, Huaping Town, Zhongjing (Yachang Orchid National Nature Reserve), 24°49.367'N, 106°24.029'E, 1341 m elevation, 29 July 2019, *Z.C. Lu et al. 20190729YC4141* (holotype: IBK; isotypes: IBK, GXMG).

## Supplementary Material

XML Treatment for Isotrema putalengense

XML Treatment for Isotrema vuquangense

XML Treatment for Isotrema yachangense
